# Supplementary material for: Are carbon emissions trading and green financial instruments synergistic? -Comprehensive quantitative research based on content analysis
Source: PLoS One. 2024 Mar 7;19(3):e0298601. doi: 10.1371/journal.pone.0298601 (PMC10919601; doi:10.1371/journal.pone.0298601)
Supplement: S1 Data — (ZIP) [file pone.0298601.s001.zip › Data/地级市Z_F工作报告.zip!/─┌├╔╣┼/│α╖σ╩╨/└┤╫╘╣1⁄2╓┌║┼í╛┬φ┐╦╩2╛▌═°í┐_2010.docx]

各位代表：

　　现在，我代表市人民政府，向大会报告政府工作，请予审议，并请市政协委员和列席会议的同志提出意见。

　　一、2009年工作回顾

　　2009年是新世纪以来我市经济社会发展最为困难的一年。面对严峻复杂形势，在市委的坚强领导下，全市各族人民坚定信心、迎难而上，化挑战为机遇、变压力为动力，改革开放和现代化建设取得新成绩。预计全市生产总值完成877亿元，增长14%；地方财政总收入82.3亿元，增长13.6%；全社会固定资产投资655亿元，增长26%；社会消费品零售总额285亿元，增长19%；城镇居民人均可支配收入12750元、农牧民人均纯收入4685元，均增长10.5%。

　　一年来，我们主要做了以下工作：

　　促进经济平稳较快发展。全面贯彻落实中央、自治区保增长、保民生、保稳定各项政策，出台应对金融危机的指导意见和促进企业正常生产的十项措施，推动经济企稳回升、持续向好。下大力促使停产半停产企业启动和恢复生产，安排企业发展基金8000余万元，取消行政事业性收费、减免缓缴困难企业社会保险费3.6亿元，因金融危机停产的168户规模以上企业全部恢复生产。千方百计扩大投资，实施3000万元以上项目374个，完成投资438亿元，黄岗铁矿180万吨选场、远联50万吨矽钢等一批重大项目建成投产。抓住国家实施积极财政政策和适度宽松货币政策的机遇，全年争取中央扩大内需资金15.1亿元，列各盟市第一位；金融机构投放贷款446亿元，较上年增加130亿元，增长41%。努力扩大消费需求，投资35亿元，实施大型商贸项目37个，新增市场建筑面积280万平方米，新建改造农家店550家；家电下乡产品销售量、销售额占自治区的四分之一，为农牧民兑付补贴1895万元。全市实施房地产开发657万平方米，完成投资130亿元，其中新城区开发面积达到120万平方米，宁城、元宝山、喀喇沁均超过60万平方米。

　　推进经济结构调整。加快科技创新，赤峰国家农业科技园区通过科技部验收初审，赤峰丹龙药业晋升为国家级高新技术企业。积极发展现代农牧业，优质高产高效农作物比重和家畜良改率分别达到70%和97%，农区畜牧业比重提高到74%，农畜产品加工转化率达到65%；新增设施农业10万亩，总面积达到56万亩；提高农牧业综合生产能力，大灾之年粮食产量达到59.2亿斤，牧业年度家畜存栏1769万头只，畜群畜种结构进一步优化。大力培育新兴产业，总投资257亿元的大唐克旗40亿立方米煤制天然气项目开工建设，国电元宝山煤制尿素、中电投右旗煤制甲醇进展顺利，九联焦化一期竣工投产，煤化工产业发展取得实质性突破；红山中凯、松山国电风机设备和翁旗鸿宇精密机械制造进展较快，喀旗亚鼎10万吨无氧铜杆开工建设，机械制造业呈现较好发展态势。积极发展现代服务业，红山物流园区扩大到5100亩，完成交易额55亿元，带动就业1万人，喀旗和美工贸园区顺利推进，松山物流园区农机交易区投入使用；美林谷滑雪场开始运营，亲王府后花园、召庙等重点旅游项目建设步伐加快；达里湖景区晋升为国家4A级旅游景区；全年接待游客520万人次、旅游收入61亿元。完成节能减排任务，单位生产总值能耗下降5.6%，化学需氧量和二氧化硫排放量分别减少0.6%和14.3%。

　　加强生态和基础设施建设。投资10.5亿元，推进重点生态建设工程，完成营造林244万亩，绿化公路397公里；落实封育禁牧等保护措施，加强水、大气污染防治，生态环境持续好转。投资40.5亿元，继续加快新城区建设，完成306国道立交桥等道路和主要街路景观绿化工程，阳光新城等一批住宅小区竣工，文博中心、青少年宫基本完工，赤峰学院附属医院新址、新城区示范初中开工建设。小新地组团征地拆迁稳步实施，桥北组团改造建设快速推进，松山北城区引资开发顺利起步。老城区服务功能进一步完善，基础建设和亮化、绿化、美化又上新水平。旗县政府所在城关镇、重点小城镇建设扎实推进，综合承载能力进一步增强。全市城镇化率达到41.5%，提高3.2个百分点。公路建设投资23.7亿元，铺筑油路1570公里，全市116个苏木乡镇实现了乡乡通油路，赤朝高速公路开工建设。铁路建设投资12.5亿元，赤锦、林白铁路全面开工，巴新铁路加快推进，赤大白铁路竣工通车。水利建设投资11亿元，三座店水利枢纽基本完工，德日苏宝冷、大石门水库实现蓄水。电网建设投资26.6亿元，500千伏华峰宁输变电工程赤峰段全线竣工，220千伏中京、新惠、西郊新扩建输变电工程投入使用，农网改造步伐加快，网架结构进一步完善。

　　深化改革开放。政府机构改革扎实推进，调整规范撤并工作部门和办事议事机构13个。事业单位改革迈出新步伐，赤峰广播电台和电视台合并，赤峰报业集团成立，新组建了赤峰农牧科学研究院。企业改革继续推进，金峰铜业、红岭铅锌矿等企业战略重组取得重要进展。农村牧区综合改革不断深化，村级公益事业建设一事一议财政奖补试点全面推开，财政资金补贴一卡通发放工作进展顺利。土地、草牧场经营权流转加快推进，元宝山和润、松山楚东等现代农牧业产业园区开始出现。集体林权制度改革全面铺开，已有45%的苏木乡镇完成产权明晰和林权证发放工作。积极扩大对外开放，加强与东北、环渤海地区的联系，推进五市一盟区域合作，成功在北京、呼和浩特等地举办系列招商活动，实施了一批新的合作项目，东北特钢、湖南有色、新疆金风等行业领军企业入驻我市。全年引进资金367亿元，增长31.7%；实际利用外资5038万美元，增长56.2%。外贸进出口在艰难形势下完成4.7亿美元，增长2.2%。

　　大力发展社会事业。元宝山区进入首批国家知识产权强县行列；内蒙古第七届农博会暨赤峰市第九届农交会完成现场交易额6970万元，影响力进一步扩大。翁旗被自治区评为两基巩固提高先进旗；全市高考本科上线率55.6%，高出全区平均水平4.2个百分点；职业教育实现突破，职普比例达到4：6；启动中小学校舍安全工程，加固改造和新建校舍51万平方米。成功举办了首届内蒙古马头琴艺术节暨第四届红山文化节；28个苏木乡镇综合文化站、7个文化信息资源共享中心、190个草原书屋投入使用。投资7000多万元，完成体育中心一期工程，实施农牧民健身工程131处。科学防控甲型H1N1流感，保护人民群众生命健康；投资2.2亿元，新建改造医疗卫生机构212所。统筹做好人口和计划生育工作，人口出生率控制在11‰。外事、侨务、人防、地震、档案、老龄、残疾人、妇女儿童和红十字等工作都取得了新成绩。

　　保障和改善人民生活。投入资金34.6亿元,市政府承诺的十件惠民实事全部完成。城镇累计新就业2.6万人，城镇登记失业率控制在4.2%。新型农村牧区合作医疗参合率提高到95.4%。敖汉旗进入国家首批新农保试点地区。城镇居民基本医疗保险参保人数达到45万，彻底解决了3.5万国有关闭破产企业退休人员医疗保险参保问题。企业退休人员养老金人均月提高125元。城乡人均月低保标准分别提高30元和15元。发放助学贷款、助学金1.2亿元，惠及6.3万名学生。完成劳务输出107万人次，劳务收入49亿元。新建廉租住房13.4万平方米、经济适用房32.9万平方米，低收入群众住房困难得到一定程度缓解。又有156个嘎查村通公路、868个自然村通广播电视。落实扶贫资金2.3亿元，扶持6.3万贫困人口实现稳定脱贫。投资1.2亿元，解决了20万人和68万头只牲畜的饮水安全问题。

　　加强精神文明和民主法制建设。以迎国庆、讲文明、树新风活动为载体，推进文明单位、文明社区、文明乡村建设，公民素质和社会文明程度稳步提升。开展机关作风建设年活动，政府工作人员工作效率和服务水平进一步提高。自觉接受人大法律监督、工作监督和政协民主监督，全年向人大及其常委会报告工作14次，向政协常委会通报情况3次，人大代表建议、意见和政协委员提案办复率100%。扎实推进五五普法。第七届嘎查村委员会换届选举工作基本结束。依法管理宗教事务，巩固民族团结大好局面。加大重点领域和专项资金审计监察力度，加强廉政风险防范管理，对小金库和工程建设领域突出问题进行专项治理，廉政建设进一步加强。高度重视信访工作，实行领导干部轮流接访制度，及时有效处置信访突出问题和群体性事件。加强社会治安综合治理，依法打击各类违法犯罪活动，维护国家安全和社会稳定。狠抓安全生产，对重点行业和领域实施了集中整治。深入推进国防动员和双拥共建，军地军民关系更加和谐。

　　各位代表，刚刚过去的2009年是极不平凡的一年。这一年，我们全力以赴应对金融危机冲击，认真落实保增长、保民生、保稳定的各项政策措施，保持了经济社会平稳较快发展。这一年，我们齐心协力抗击历史罕见特大旱灾，认真贯彻温家宝总理亲临视察时的重要指示，组织广大干部群众抗灾自救，取得了抗旱减灾的重大胜利；这一年，我们果断处置新城区水污染、赤峰制药集团氨气泄露等突发事件，深刻汲取教训，切实维护了人民群众生命健康和社会大局稳定。

　　回顾过去一年的工作，我们深刻体会到，越是困难的时候，越要自觉贯彻落实科学发展观，坚持发展第一要务不动摇，用发展的办法解决前进中的问题。越是困难的时候，越要坚定信心，提振士气，凝聚力量，充分发挥各级干部和广大人民群众的积极性和创造性，形成攻坚克难的强大合力。越是困难的时候，越要解放思想，开拓创新，在变化中捕捉和把握机遇，在逆境中培育和发现有利因素，牢牢掌握工作主动权。越是困难的时候，越要高度关注民生，真心实意为群众办实事、做好事、解难事，促进社会和谐稳定。实践证明，无论遇到多少艰难险阻，只要我们坚定不移地走科学发展之路，同心同德，团结奋进，就一定能够不断夺取经济社会发展的新胜利！

　　各位代表，过去一年的成绩来之不易。这些成绩的取得，是市委统揽全局、正确领导的结果；是人大代表、政协委员加强监督、大力支持的结果；是全市上下齐心协力、扎实工作的结果。在此，我代表市人民政府，向全市各族人民，向所有关心支持赤峰发展进步的同志们、朋友们，致以崇高的敬意和衷心的感谢！

　　在肯定成绩的同时，我们也清醒看到，我市经济社会发展还存在不少困难和问题。主要是：经济总量小，人均水平低，地区发展不平衡；经济回升基础不够稳固，部分企业生产经营困难，效益不明显；经济结构不够合理，产业链条短，产业层次不高，多元发展的产业体系尚未完全建立；民间资本活力不足，中小企业发展不快；农牧业基础薄弱，抗御自然灾害能力不强；城镇化水平较低，城市基础建设滞后，管理水平不高，吸纳和辐射带动能力较弱；财政减收增支因素增多，收支矛盾十分突出；民生领域还存在不少亟待解决的问题，城乡居民增收缓慢，部分群众生活比较困难；一些政府部门和工作人员大局意识、责任意识、服务意识需进一步增强，工作作风需进一步转变。对于这些问题，我们一定采取有力措施，认真加以解决，不辜负人民的期望和重托。

　　二、2010年主要任务

　　今年是实施十一五规划的最后一年。做好今年工作，全面完成十一五规划目标任务，对夺取应对金融危机全面胜利、保持经济平稳较快发展、顺利启动实施十二五规划，具有十分重要的意义。

　　当前，金融危机的影响仍然存在，面临的形势依然复杂而严峻。但我市经济社会发展的基本面和长期向好趋势没有改变。中央保持宏观经济政策的稳定性和连续性，继续实施积极的财政政策和适度宽松的货币政策，加大对欠发达地区支持力度，这是我们增加投资、扩大总量的良好机遇；随着国内外经济复苏步伐加快和我国工业化、城镇化快速推进，市场对能源、原材料、劳动力需求日趋旺盛，这是我们发挥优势、加快发展的有利条件；我市有较好的产业基础，有色金属、能源、食品加工等优势产业快速发展，煤化工、风电等新兴产业迅速崛起，建材、纺织、制药等传统产业焕发生机，这是我们持续发展、长远发展的坚实基础；我市有良好的区位优势，随着一批公路、铁路、电力、水利等基础设施建成，发展后劲显著增强，这是我们厚积薄发、快速赶上的有力支撑；赤峰干部群众素质较高，具有顽强拼搏、百折不挠的精神，谋发展、求发展的愿望十分强烈，特别是在各种重大考验中积累了宝贵经验，这是我们跨步前进、振兴崛起的根本保证。只要我们发挥优势，趋利避害，坚持发展不动摇，扎实工作不松劲，就一定能够战胜各种艰难险阻，迎来新一轮经济社会大发展。

　　今年政府工作的总体要求是：深入学习实践科学发展观，全面贯彻落实市委五届十二次全委会议精神，坚持发展是硬道理的战略思想不动摇，以保持经济平稳较快增长、做大经济总量为首要任务，调整经济结构，转变发展方式，推进持续发展；以改善发展条件、促进城乡统筹为重要手段，加强生态和基础设施建设，加快新型工业化、农牧业产业化、城镇化进程，推进协调发展；以保障和改善民生为根本目标，发展社会事业，维护团结稳定，努力增收富民，推进和谐发展，全面开创改革开放和现代化建设新局面，以优异成绩迎接自治区两个文明建设经验交流会在我市召开。

　　主要预期目标是：地区生产总值增长15%左右，突破1000亿元；地方财政总收入增长18%左右，力争达到100亿元；城镇居民人均可支配收入和农牧民人均纯收入分别增长11%；全社会固定资产投资增长25%；社会消费品零售总额增长18%。这些目标综合考虑了可能与需要、当前与长远，既突出了能快就不要慢的思想，以充分调动社会各方面的积极性、创造性；又坚持从实际出发，实事求是，注重速度、质量、结构、效益、环境相统一，体现了积极不保守、客观不浮夸的原则。

　　（一）提升产业发展水平，加快新型工业化进程

　　大力培育特色产业。抓好有色金属、能源、食品加工等优势产业，培育化工、非金属、清洁能源等新兴产业，提升建材、纺织、制药等传统产业，构建竞争能力强、发展后劲足、富有特色的工业体系。全年实施投资1000万元以上工业项目197项，完成投资280亿元。抓好国维矿业3万吨铜钼选场、中色10万吨锌冶炼、亚鼎10万吨无氧铜杆等一批有色金属采选、冶炼和深加工项目，继续做大做强有色金属产业。做好中电投大板电厂2×60万千瓦机组投产前的各项准备工作，力争早日并网发电，开工建设中电投松山区2×30万千瓦热电机组，推进大唐克旗超超临界机组等项目前期工作，大力发展能源产业。引进知名龙头企业，发展粮食、蔬菜等农产品和肉牛、肉羊、生猪等畜产品加工，支持现有企业扩大规模、提升实力，壮大食品工业。加快大唐煤制天然气、中电投煤制甲醇、国电煤制化肥等煤化工项目建设，尽快把化工产业培育成新的支柱产业。加强与科研机构和大企业合作，继续开展非金属资源勘查，开发新型材料和非金属系列产品，发展非金属产业。努力解决好风电上网的瓶颈制约问题，全年新增风电装机60万千瓦，加快阳光科技1000吨光伏材料建设，推进中国巨力太阳能发电前期工作，大力发展清洁能源产业。抓好远航、巴彦包特、华林等水泥企业重组扩建，壮大建材产业。推动赤峰毛业等纺织企业加快实施技术改造，提升产品档次，提高市场占有率。扶持制药企业发挥产业基础较好和市场网络健全优势，加大新产品研发力度，开拓市场，实现振兴。

　　全力抓好园区建设。推进工业园区和工业集中区水、电、路建设，改变园区基础设施薄弱状况，推动工业企业和特色产业入园发展。扩大土地储备，为项目建设和企业入驻留足空间。规划园区产业布局，明确功能定位，为园区规范有序发展奠定基础。编制赤峰经济开发区总体发展规划，统筹抓好红山、松山和元宝山三个园区基础设施、项目建设和招商引资，做大开发区经济总量。按照产业配套、产业延伸、循环发展的原则，建设一批机械制造、服装加工、食品加工等专业特色园区，促进产业集群化发展。

　　加快发展中小企业。落实国家、自治区各项政策措施，在准入、融资、土地、税收等方面加大扶持力度。建立中小企业发展基金，完善适合中小企业特点的评级和授信制度，健全以赤峰恒德担保公司为龙头的信用担保体系，力争为中小企业融资20亿元以上。加快发展小额贷款公司，筹建地方性金融机构，拓宽中小企业融资渠道。推动股份制银行、邮储银行等金融机构开发中小企业信贷产品，满足中小企业融资需求。降低准入门槛，简化审批手续，鼓励支持创办中小企业。

　　加大矿产资源勘探整合开发力度。以白音诺尔、大井子等25个矿区探矿权、采矿权整合为重点，整顿和规范矿产资源开发秩序，确保年内完成全部整治任务。加大探矿投入，加快勘探步伐，尽快摸清全市矿产资源状况。坚持政府引导、市场运作，综合运用经济、法律和必要的行政手段，推动资源向投资规模大、技术水平高、市场前景好和在我市实施深加工的大企业、大集团集中，提高产业集中度。建立资源开发生态补偿机制，依法落实环境保护、治理、恢复的责任，决不允许把环境污染和生态破坏的代价留给社会、留给子孙后代。

　　（二）发展现代农牧业，促进农牧业增效农牧民增收

　　优化农牧业结构。实施百亿斤粮食生产能力建设工程，推进农业综合开发和土地整治，稳定粮食播种面积，扩大优质高产高效作物种植比重，提高单产水平，粮食产量正常年景达到70亿斤以上。推进百万亩设施农业建设，抓好规划调地、资金扶持、技术服务、市场开拓等关键环节，推广公司化经营、反租倒包等新形式，确保年内新增设施农业10万亩，继续保持自治区领先水平。实施百万亩节水灌溉工程，科学规划，集中资金力量，推进规模建设，年内新增喷灌、滴灌、渗灌等节水灌溉面积30万亩以上。推进百万头肉牛育肥工程，制定肉牛育肥规划，抓好专业村和养殖小区建设，推广西门塔尔等优质品种，全市育肥牛出栏60万头，三年内达到100万头。推广应用生态安全生产技术措施，发展绿色、有机、无公害农畜产品。

　　推进产业化经营。扶持塞飞亚、草原兴发、东方万旗、蒙都肉羊、新新杰蔬菜加工、荣兴堂药业等企业扩大规模，培育品牌，进一步做大做强。促进冷山糖业、宏发肉牛肉羊屠宰、中鹿鹿产品深加工、科然生物大豆深加工项目尽快达产达效。加快雨润200万口生猪加工、万顺达120万吨玉米淀粉等项目建设，发挥辐射带动作用。支持上京农畜产品加工园区、玉龙食品工业园区加快发展，推动龙头企业向园区集中。大力发展农牧民专业合作组织，通过农牧户参股、公司提供资金技术支持等方式，密切企业与农牧户合作关系，加快建设肉牛、肉羊、生猪以及蔬菜、玉米、杂粮、药材等专业村和标准化生产基地，解决龙头企业原材料不足问题。

　　加强社会化服务体系建设。健全农牧业科技服务体系，创新利益联动机制，加大先进适用技术推广力度，提高科技对农牧业生产贡献率。完善农畜产品市场体系，支持农畜产品主要产地批发市场、集贸市场建设，抓好市场信息服务，引导流通企业建立直接采购基地。健全农畜产品质量安全体系，抓好动物疫病防控、植物保护和质量检测，保障农畜产品质量安全。完善金融服务体系，鼓励支持金融机构发展农村牧区小额信贷和微型金融服务，推广农牧民信用互助模式，扩大政策性农牧业保险范围。

　　增加农牧民收入。改善生产条件，优化种养业结构，提升农畜产品产量和质量，发挥农牧业生产对农牧民增收的主渠道作用。抓好生态观光农牧业，发展特色农家乐、牧家乐、林家乐和多种经营活动，增加经营性收入。引导鼓励农牧民向城镇二、三产业转移，提高工资性收入比重。落实好各项惠农、惠牧补贴政策，增加农牧民政策性收入。大力发展劳务经济，劳务输出稳定在100万人次。实施项目扶贫、科技扶贫、社会扶贫工程，促进6.3万贫困人口稳定脱贫。

　　（三）大力发展服务业，满足人民群众消费需求

　　改造提升商贸服务业。统筹规划大型商场、高档宾馆和娱乐服务设施建设，加快中心城市商圈及旗县大型商贸项目推进步伐，实施航宇商务大厦、印象红山商务中心、维多利商务广场、阳光时代广场等大型商贸工程，推动金钰大都会、众联广场尽快投入运营，吸引全市乃至周边地区居民购物消费。发展配送中心等新兴业态，推行连锁、代理等经营方式，提高商贸服务业的档次和水平。推进万村千乡市场工程和新网工程建设，农家店覆盖所有行政村，农资配送率达到95%以上。做好家电、汽车、摩托车下乡工作，鼓励农牧民购置农机具、农用车、家用电器等耐用消费品，拓展农村牧区消费市场。

　　做大做强物流业。统筹做好物流园区发展规划，进一步明确各园区发展方向和功能定位，促进物流业健康发展。推进红山物流园区三期建设，完成赤通高速北出口、铁路专用线等重点配套工程，抓好汽修汽配城、石油仓储库等重点项目，完善集装箱中转、海关商检服务功能，丰富经营业态，年交易额达到80亿元以上。加快松山物流园区建设，扩大土地储备，完善基础设施，建设五金机电、农机交易、农副产品市场等功能区。加快喀旗和美建材城建设，推动红星美凯龙家居品牌入驻，确保8月份运营；实施和美商贸城、餐饮娱乐城、文化产业园等项目。建设大板物流园区，打造煤炭物流集散地。推动各旗县区结合自身条件和产业优势，建设功能不同的物流园区和配送中心，提高辐射带动能力。

　　提升旅游业发展水平。高起点编制旅游景区、景点发展规划，统筹抓好餐饮、住宿、购物、娱乐设施建设，科学谋划旅游精品线路。加快阿斯哈图、达里湖、乌兰布统、美林谷、黑里河等重点景区景点建设，搞好克旗、宁城、敖汉热水整体开发。在央视等媒体、重点旅游客源地和高速公路、省际大通道、城区主要道路两侧进行全方位、多角度宣传，提高赤峰旅游知名度。研发一批民族特色突出、附加值高的旅游产品，抓好市场营销，促进旅游消费。加强旅游培训，强化旅游监管，提升服务水平。全年接待游客达到580万人次，实现旅游收入74亿元。

　　加快发展金融业。落实银政合作协议，推动金融企业扩大信贷规模，支持地方经济发展。完善激励机制，鼓励创新服务，引导支持金融机构贷款向三农三牧和中小企业倾斜。建立健全银政、银企合作对接平台，及时向金融系统发布重大基础设施、重点生产建设项目、重要社会事业项目和民生工程等方面的信息，金融机构信贷投放力争增长20%以上。筹建农村合作银行，组建敖汉、宁城、喀喇沁村镇银行，支持各金融企业在旗县区设立分支机构。新增小额贷款公司12家，总数达到32家。做大做强担保机构实力和规模，推进担保品种、抵押方式创新。力争引进2 - 3家股份制商业银行，健全多层次的金融服务体系。加强诚信赤峰建设，做好金融风险防范工作，维护金融安全稳定。

　　（四）加快城镇化进程，促进城乡协调发展

　　加大中心城市建设力度。围绕建设百万人口区域性中心城市，高标准修编城市总体规划，统筹抓好新城区、红山城区、松山城区和元宝山城区开发建设，打造环境整洁、宜居舒适、生态文明的现代化城市。投资40亿元，加快小新地组团基础设施建设，实施体育中心二期工程，抓好房地产规模开发；完善八家组团配套服务功能，加大住宅小区、学校、医院和商贸服务设施建设，解决好供水、供电、供热、排水等问题。投资10亿元，推进红山城区桥北组团开发，实施道路、桥梁、管网等基础设施和住宅小区建设，建成樟子松公园、滨河景观带。投资5亿元，加快老城区改造，实施东城大街综合治理，建设清河大桥和步行街大桥，推进供水、供热管网改造。投资20亿元，搞好松山北城区一期征地拆迁，开工建设友谊大街等基础设施。投资10亿元，实施元宝山城区如意河改造开发、平庄市民广场等工程。加快国家森林城市创建步伐，抓好重点区域、关键环节绿化美化。中心城市新增5万人，总人口达到89万。

　　加快旗县政府所在城关镇和重点小城镇建设。完成旗县政府所在城关镇和部分重点小城镇规划修编。加快乌丹、锦山、天义、新惠新区开发，抓好林西旧城改造、林东西南组团开发和大板城区出口、天山西河治理、经棚滨河带状公园等重点项目建设，提高旗县政府所在城关镇综合承载能力。每个旗县选择3至5个基础条件好，区位、产业、规模优势突出的小城镇，制定融资、拆迁、土地储备等支持政策，加快市政、园林、道路等基础设施建设，打造经济活跃、特色鲜明、风格各异的小城镇。以推进社会主义新农村新牧区示范村建设为重点，实施改水改厕、户用沼气、垃圾处理等工程，完善村庄基础设施，抓好绿化、美化、净化工作。完成8228户农村牧区危房改造，改善农牧民居住条件。

　　加强城镇经营管理。发挥市和旗县区城镇建设融资平台作用，拓宽融资渠道，缓解城市建设资金压力。加大土地收储力度，严格实行经营性用地招拍挂，提高土地收益。采取参股、冠名、拍卖、转让等方式，吸引外来资本和民间资金参与城市建设，实现城市滚动发展。整合城市管理资源，完善综合执法体制。强化市政、园林设施管护，促进公用设施正常高效运转。推进数字化城市试点建设。加强物业管理，提高社区管理和服务水平。发展城市公交，加强出租车管理。强化规划监察，加大违法违章建筑查处力度，确保城市建设依法、合规、有序。开展城镇市场、交通、环境专项整治，解决占道经营、交通混乱、烟尘污染、噪声扰民等问题。

　　提升城镇人口吸纳能力。加大房地产开发建设力度，全市房地产开发面积700万平方米以上，其中中心城区达到260万平方米，旗县政府所在城关镇不少于30万平方米，重点小城镇不少于2万平方米。加快保障性住房、回迁安置房建设，增加普通商品房供给，满足不同层次住房需求。大力发展旗县政府所在城关镇和重点小城镇二、三产业，吸引农村牧区人口向城镇转移。放宽城镇户籍限制，解决进城务工人员子女入学、社会保障等问题，引导更多农牧民在城镇落户就业。全市城镇化率提高3.5个百分点，达到45%。

　　（五）加强生态和基础设施建设，提高发展保障能力

　　进一步改善生态环境。实施好京津风沙源治理、退耕还林等重点生态建设与保护工程，完成营造林面积233万亩。加快翁旗东部沙地治理，实施穿沙公路、林果基地、引水入沙等防沙治沙工程，完成综合治理70万亩。推广公司制、股份制、合作制等形式，完善利益导向机制，调动各类主体投资生态建设的积极性。把加强生态建设与富民增收结合起来，大力培育木材加工、绿色食品加工、森林生态旅游、特色养殖、生态能源林、种苗花卉六大产业，促进生态、经济、社会效益相统一。强化封育禁牧、舍饲圈养、生态移民等保护措施，严厉打击非法开荒、毁林毁草等行为，巩固生态建设成果。

　　推进交通基础设施建设。加快巴新、赤锦、林白铁路建设，力争早日投入运营。做好征地拆迁等基础性工作，确保集通二线、白浩、叶赤铁路扩能改造开工建设。积极推进赤峰至凌源高速铁路前期工作，认真谋划克旗至辽宁绥中铁路。完成赤朝高速公路主体工程和林西镇、林东镇至省际通道、经棚至热水一级公路建设，确保赤承高速公路、赤峰至天义、安庆至平庄一级路年内开工。建设通村公路17条、210公里。实施赤大高速、赤通高速、环城高速、赤元一级公路和赤大白铁路沿线绿化、美化及环境整治工程。改造赤峰玉龙机场，提高客运能力。

　　完善电力、水利基础设施。加快500千伏华峰宁输变电配套工程和南部电网工程建设，提高供电保障能力。完成220千伏锦山、林西、城东一次变工程，开工建设220千伏经西、金厂沟梁、绍根输变电工程，实现每个旗县区至少拥有一座220千伏输变电站目标。积极推进500千伏风电专送通道和赤峰至华东特高压直流输电线路前期工作，力争早日列入国家规划并启动实施。抓好大型灌区续建配套和节水改造项目，推进末级渠道和田间工程配套，尽快建成完善的节水灌溉体系。完成规划内全部病险水库除险加固任务，确保三座店水利枢纽和德日苏宝冷、大石门水库竣工，加快推进东台子水库前期工作。

　　强化节能减排。抓好有色金属、电力、建材等重点行业节能降耗，坚决淘汰落后生产工艺。开发推广循环利用、节约资源和治污减排先进技术，发展循环经济和低碳产业。抓好赤峰再生资源加工利用循环经济园区建设。完成中心城区污水处理厂三期扩建和中水回用工程，日处理污水达到16万吨、生产中水5万吨。加快红山经济开发区、元宝山镇污水处理厂建设，完善配套设施，加强监督管理，确保已建成的污水处理厂正常运行。推进建筑节能，完成120万平方米供热分户计量试点工作，争取进入国家可再生能源试点城市。强化节能减排责任落实，二氧化硫排放和化学需氧量分别控制在18万吨和1.6万吨以内，完成十一五节能减排任务。

　　（六）推进改革开放，增强发展活力动力

　　深化重要领域改革创新。推进村级公益事业改革，一事一议财政奖补覆盖所有旗县区。加快土地和草牧场流转，完善流转制度，搭建服务平台，提供便捷高效的流转服务。推进集体林权制度改革，基本完成明晰产权和林权证发放工作。深化水利工程管理体制改革，发展农牧民用水协会组织。抓好文化体制改革，理顺电影管理职能，创新文化事业单位内部管理机制。深化医药卫生体制改革，建立基本医疗保障和基本药物制度，健全基层医疗卫生服务体系，推动公立医院改革试点。在市本级行政事业单位全面推行公务卡制度。稳步实施城市公用事业价格改革。搞好政府机构改革和事业单位绩效工资改革。

　　进一步扩大对外开放。抓住发达地区产业加快转移机遇，规划建设承接产业转移园区，完善服务功能和配套设施，吸引更多企业入驻。加强与北京、河北、辽宁、天津、山东等环渤海地区合作，积极承接以纺织、服装、制鞋为主的劳动密集型产业，有色金属、能源、化工为主的精深加工产业，矿山机械、小五金、零配件加工为主的机械制造产业和整体配套产业转移，提升发展层次。提高农牧业、服务业和社会事业领域对外开放水平。加强与进驻我市大企业、大集团战略投资合作，实施一批大项目、好项目。全年引进资金458亿元，增长25%。支持外贸企业优化出口结构，开拓外贸市场，完成进出口总额4.8亿美元，增长3%。

　　大力发展非公有制经济。按照非禁即入原则，拓宽非公有制经济发展空间，发挥其繁荣经济、活跃市场、扩大就业作用。引导非公有制企业加强技术创新、体制创新和管理创新，支持本地企业与市外企业搞好资本和技术联合，做强主业，做优品牌，做大规模，提高核心竞争能力。优化经济发展环境，搭建政企对接平台，及时倾听企业合理诉求，帮助解决融资、技术、品牌等方面难题。加强企业家队伍建设，组织开展学习交流和培训活动，为非公有制经济持续发展提供人才保障。

　　（七）发展社会事业，提高公共服务水平

　　推动科技进步。抓好科技创新平台建设，确保3家企业晋升为国家级高新技术企业，3家企业通过自治区级企业研发中心认定。实施工业强市科技引领工程，支持医药、食品、非金属材料、能源、有色金属等领域开展技术攻关和产品研发。加快5个农牧业示范园区和9个农业特色产业化基地建设。完成农牧民科技培训80万人次。推进全国科普示范县创建，搞好知识产权试点市建设。办好自治区第八届农博会暨赤峰市第十届农交会。抓好人才引进、培养和使用，为经济社会发展提供智力支撑。

　　优先发展教育事业。加大教育投入力度，推动公共教育资源配置向农村牧区倾斜，促进义务教育均衡发展。抓好两基巩固提高，继续推进学校布局调整，优化教育资源配置。普及高中阶段教育，扩大中等职业教育招生规模，实现职普比例大体相当。加强中等职业学校基础能力建设，完成赤峰第一职业中专、喀旗建筑职业学校、宁城职教中心搬迁。优先发展民族教育，重视特殊教育和学前教育，鼓励和支持社会力量办学。推动赤峰学院、内蒙古交通职业技术学院调整优化专业结构，培养更多符合区域经济社会发展的专业人才和技能型实用人才。做好内蒙古纺织工业学校申报高等职业技术院校工作。切实抓好中小学校舍安全工程建设，保证质量、安全施工、加快进度，把学校建成最安全、家长最放心的地方。

　　提高人民健康水平。加快赤峰学院附属医院新址建设，完成市中蒙医院和红山区、翁旗、右旗医院病房楼及宁城县医院门诊楼建设，实施阿旗、松山区医院整体搬迁。加强农村牧区医疗卫生服务网络、城市社区卫生服务机构和基层医疗卫生队伍建设，提高基层医疗卫生服务水平。支持中蒙医事业加快发展。加强甲型H1N1流感及各类传染病防治，提高突发公共卫生事件应急处置能力。大力开展爱国卫生运动，巩固国家卫生城市创建成果。抓好生育关怀和出生缺陷预防，提高出生人口素质，人口出生率控制在12‰以内。广泛开展全民健身运动，提高城乡居民健康水平。积极参加自治区第十二届运动会，做好自治区第十三届运动会申办工作。

　　发展文化事业和文化产业。改善公共文化服务基础设施，完成43个苏木乡镇综合文化站建设，确保文博中心投入使用。继续实施广播电视村村通、文化信息资源共享和农村牧区电影放映工程，满足群众文化生活需求。加强文物和非物质文化遗产保护，做好二道井子、魏家窝铺遗址保护、发掘和展示工作。完成辽陵及奉陵邑保护规划，实施辽上京遗址公园开发，启动辽代大遗址群申报世界文化遗产工作。加强辽代历史文化宣传，打造辽文化品牌。加快文化产业园区建设，完成巴林石文化创意产业园工程，搞好巴林石创意、雕刻、展览和营销。大力发展演艺娱乐、文化创意、文博会展、图书印刷等特色文化产业。积极发展民间演出团体，创作精品演出曲目，丰富群众文化生活。加强网络文化建设和监督管理，净化网络环境。办好第五届红山文化节。

　　（八）保障和改善民生，维护社会和谐稳定

　　千方百计扩大就业。大力发展服务业、劳动密集型产业、中小企业和非公有制经济，创造更多的就业岗位。突出抓好高校毕业生就业工作，加快建设一批投资少、见效快的大学生创业园和创业孵化基地，鼓励支持高校毕业生自主创业、到基层就业。发挥政府投资和重点项目建设带动就业作用，组织农民工参与项目和基础设施建设，引导农民工有序流动。开辟公益性就业岗位，帮助城镇就业困难人员、零就业家庭和灾区劳动力就业。加快创业型城市建设，在市场准入、财税金融、经营用地等方面提供便利和优惠，鼓励更多劳动者成为创业者。加强就业信息发布、就业指导和职业培训，改进就业公共服务。

　　完善社会保障体系。做好基本养老保险省级统筹工作，落实基本养老保险关系跨省转移接续政策，保障流动就业人群权益。实施城镇基本医疗保险市级统筹，制定公务员医疗保险补助办法。完善社会救助制度，发挥残联、红十字会、慈善总会、社会救助站作用，开展多种形式的社会救助活动，努力解决低收入群体生活困难问题。做好非公有制经济从业人员、农民工、被征地农牧民、灵活就业人员和自由职业者参保工作，城镇和农村牧区低保实现动态管理下的应保尽保。加强社会保障基金监管，确保基金安全。

　　推进公共服务均等化。优化财政支出结构，重点向就业、社会保障等领域和社会困难群体倾斜。落实城乡免费义务教育，对牧区中等职业学生实行免费教育，加大助学贷款、助学金补助力度。坚持公共医疗卫生公益性质，调动广大医务人员积极性，实施万名医师支援农村牧区卫生工程。扩大免费公共卫生服务范围，支持偏远地区嘎查村卫生室建设，逐步解决看病难、看病贵问题。完成妇女儿童十一五规划目标任务。做好老年人、残疾人工作，加强社会福利院、儿童福利院、敬老院建设，提高社会福利保障水平。

　　全力维护社会稳定。扎实推进平安建设，加强社会治安综合治理，依法严厉打击各类违法犯罪活动，维护国家安全和社会稳定。全面落实信访工作责任制，切实解决群众反映的突出问题，努力把矛盾化解在基层、解决在萌芽状态。加强应急管理体系建设，完善社会稳定预警机制，有效预防、妥善处置各类群体性事件。加强食品卫生监管，确保食品卫生安全。强化安全生产监管，加大矿山、烟花爆竹、危险化学品、交通运输、建筑施工等重点行业领域隐患排查和专项整治力度，坚决遏制重特大安全事故发生。完成五五普法规划任务。深入开展双拥共建活动，争创全国双拥模范城七连冠。全面贯彻党的民族宗教政策，依法管理民族宗教事务。做好人防、气象、防震减灾等工作。

　　（九）认真解决群众切身利益问题，让群众得到更多实惠

　　坚持不懈为群众做好事、办实事、解难事，是政府的重要职责。今年我们要继续集中力量办十件人民群众最关注、最直接、最现实的事情，让更多群众受益。

　　1、统筹做好就业再就业工作。筹措资金7400万元，开发公益性岗位3000个，培训下岗失业人员1.8万人，培训农村牧区劳动力27万人，全年城镇累计新就业2.8万人。

　　2、扩大养老保险覆盖面。农村牧区新型养老保险参保人数增加2.5万人，达到11.5万人；城镇企业职工养老保险参保人数增加1.2万人，达到26.8万人。

　　3、提高社会保障标准。筹措资金6.4亿元，城镇低保人均月提高20元以上，9.5万人受益；农村牧区低保人均年增加120元，23.2万人受益；五保供养标准人均年提高200元，覆盖1.8万五保对象；企业退休人员基本养老金人均月提高120元。

　　4、提高新农合补助水平。新农合人均筹资水平由100元提高到150元，住院补偿封顶线由3.5万元提高到4万元，实行门诊、住院、大病统筹补偿，让更多农牧民受益。

　　5、实施全民健康服务工程。安排资金1600万元，用于补种乙肝疫苗、白内障患者手术补助、农村牧区产妇住院分娩等公共卫生项目。安排资金2500万元，推进一杯奶健康工程，使农村牧区3.5万孕期妇女受益。为每位城乡居民建立健康档案，提供15元的基本公共卫生服务。

　　6、抓好中小学校舍安全工程。筹措资金22.4亿元，对392所中小学校舍进行新建改造，新建校舍117万平方米，加固校舍149万平方米，完成校安工程总量的60%。

　　7、继续推进保障性住房建设。筹措资金5.4亿元，建设经济适用房20万平方米、廉租住房13.2万平方米。

　　8、实施农村牧区人畜饮水安全工程。筹措资金1.3亿元，建设人畜饮水工程346处，解决22万人、49万头只牲畜饮水安全问题。

　　9、提高中心城区供水保障能力。筹措资金1.8亿元，改造旧水源地，开辟新水源地，整治周边环境，完善供水设施，强化监管措施，确保中心城区广大市民安全用水。

　　10、加快管道天然气建设。完成中心城区天然气主管道铺设，完善相关配套设施，使2万户居民用上方便、清洁、价廉的管道天然气，力争3年时间，覆盖中心城区70%以上住户。

　　（十）加强政府自身建设，提高驾驭经济社会发展能力

　　坚持依法行政。严格按照法定权限和程序行使权力、履行职责，不断提高依法行政水平。贯彻落实行政许可法，推进行政审批制度改革，减少行政许可和审批事项。坚持重大事项向人大报告、向政协通报制度，认真办理人大代表议案、建议和政协委员提案。主动听取民主党派、工商联和无党派人士意见，自觉接受人民群众监督和新闻舆论监督。落实重大决策公开咨询、公开听证和社会公示制度，让人民群众广泛参与重大决策和公共事务管理，确保权力在阳光下运行。

　　提高行政效能。加强电子政务和行政审批服务中心建设，积极推进网上审批和一站式服务，减少审批环节，规范审批行为。强化绩效考核和效能监察，下力量解决一些政府工作人员责任心不强、作风散漫、效率低下等问题。理顺和规范部门职责权限，完善部门协调配合机制，解决职能交叉、权责脱节、政出多门等问题。严肃查处行政不作为、有令不执行等影响行政执行力的行为，确保令行禁止、政令畅通。加强应急管理，提高处置群体性、突发性事件能力。

　　切实转变作风。政府全体工作人员都要强化发展意识，勇于克服困难、敢于承担责任、善于解决矛盾，进一步解放思想，开拓进取，创造性地开展工作。要坚持把群众利益放在首位，深入调查研究，密切联系群众，关心群众疾苦，倾听群众呼声，为群众排忧解难。要继续精简会议、文件，切实改进文风、会风。要严格控制一般性财政支出，减少招待、公车、出国等费用，坚持从简办一切事业。

　　坚持廉洁从政。抓好惩防体系建设，针对腐败现象易发多发的领域和环节，进一步加强制度建设和权力制约，从源头上防治腐败。加大审计和行政监察力度，加强工程建设、房地产开发、土地管理、矿产资源开发、社会保障等领域监管，严肃查处违法违纪行为。各级政府都要率先垂范，自警自励，勤勉尽责，以实际行动建设人民满意政府。

　　各位代表！赤峰正处在加快发展的重要时期，抢抓新机遇，应对新挑战，推进大发展，是时代赋予我们的重大责任和神圣使命。让我们在市委的正确领导下，团结拼搏，锐意进取，为实现经济社会又好又快发展、开创赤峰更加美好未来而努力奋斗！
